# The Human Gut Phage Community and Its Implications for Health and Disease

**DOI:** 10.3390/v9060141

**Published:** 2017-06-08

**Authors:** Pilar Manrique, Michael Dills, Mark J. Young

**Affiliations:** 1 Department of Microbiology and Immunology, Montana State University, Bozeman, MT 59717, USA; pilar.manrique.ronquillo@gmail.com (P.M.); michael.dills@msu.montana.edu (M.D.); 2 Department of Plant Sciences and Plant Pathology, Montana State University, Bozeman, MT 59717, USA

**Keywords:** gut microbiome bacteriophages, gut microbiome phages, gut prophage reservoir, healthy gut phages

## Abstract

In this review, we assess our current understanding of the role of bacteriophages infecting the human gut bacterial community in health and disease. In general, bacteriophages contribute to the structure of their microbial communities by driving host and viral diversification, bacterial evolution, and by expanding the functional diversity of ecosystems. Gut bacteriophages are an ensemble of unique and shared phages in individuals, which encompass temperate phages found predominately as prophage in gut bacteria (prophage reservoir) and lytic phages. In healthy individuals, only a small fraction of the prophage reservoir is activated and found as extracellular phages. Phage community dysbiosis is characterized by a shift in the activated prophage community or an increase of lytic phages, and has been correlated with disease, suggesting that a proper balance between lysis and lysogeny is needed to maintain health. Consequently, the concept of microbial dysbiosis might be extended to the phage component of the microbiome as well. Understanding the dynamics and mechanisms to restore balance after dysbiosis is an active area of research. The use of phage transplants to re-establish health suggests that phages can be used as disease treatment. Such advances represent milestones in our understanding of gut phages in human health and should fuel research on their role in health and disease.

## 1. Introduction

The human gut is home to one of the most densely populated known microbial communities known and is required for human health. Human cells, microbial cells (which include bacteria, archaea, protozoa, and fungi), and their viruses coexist in a dynamic equilibrium in healthy individuals [1]. In the gut, the most abundant members of the microbiome are bacteria and their bacteriophages [1]. Even though individuals have relatively distinct gut microbial communities at lower taxonomical levels [2], a general concept of a healthy microbiome has emerged [1,3]. The gut microbiome of healthy adult individuals is a succession of “steady-states” characterized by high resilience (ability to return to an equilibrium state after a stress-related perturbation; Box 1) [3,4,5], a conserved functional profile independent of taxonomic membership [3,6,7,8], and a common structure at the phyla level [3,9], in which Firmicutes and Bacteroidetes are the dominant members. A dysbiotic state, characterized by an altered microbiome community structure that departs from a balanced ecology, has been correlated with multiple diseases and conditions [1,3,9,10,11]. Currently, research efforts are directed towards understanding the processes that lead to gut dysbiosis and how equilibrium can be re-established in order to maintain health.

Since the discovery of phages by Felix D’Herelle 100 years ago, human-associated bacteriophages have been largely studied from a disease perspective in the context of single pathogenic bacterial species [12], either as treatment to eliminate a bacterial disease or as pathogenesis-determinant vectors for bacteria [13,14]. However, bacteriophages are not always associated with disease. They are the most abundant entities on the planet, and are considered one of the major drivers of the structure and function of microbial communities [15]. The increased appreciation for the influence of bacteriophages in microbial communities has spurred extensive investigation into the role that phages play in the human microbiome, and how they can ultimately contribute to health or disease [16].

It has been proposed that a shift in the gut bacteriophages community composition can contribute to the shift from health to disease [11,17,18]. We refer the reader to excellent reviews on the subject [17,18,19,20]. However, the specific role and effect of phages beyond correlation in health and disease remains to be determined. Despite advances in understanding the positive contribution of bacteriophages to human health [20], the detailed phage-microbe dynamics that take place during healthy “steady-states” of the microbiome remain elusive, and deserve further investigation.

In this review, we describe our current understanding of the gut phage community. We examine the role of phages in shaping the microbiome structure from birth to adulthood. We highlight some of the potential dynamics that can contribute to a resilient and balanced coexistence of bacteria and their phages during “steady-states” of a healthy adult microbiome. Specifically, we focus on the balance between lysis and lysogeny and its potential impact on health. We also revisit the early ideas of phage therapy, albeit applying them at the whole community scale, and explore the possible use of bacteriophages to recover the necessary structure of the microbiome and reestablish health.

## 2. Outcome of Phage–Bacteria Interactions

The fate of a bacterial cell upon infection with a phage can be either cell death (lysis), or temporary symbiosis (lysogeny, chronic infections). Lytic phages (also known as virulent phage), introduce their viral genome into the cell, undergo replication, and lyse their host cell to release the progeny virions. Lysogenic phages (also known as temperate phage), after introducing their DNA into the host cell, can either undergo lytic replication or integrate their DNA in the bacterial chromosome and replicate passively with their host without producing any virions [21]. The integrated phage is known as a prophage. In response to certain triggers, the prophage can become activated and switch to the lytic cycle. In pseudolysogeny, the DNA is maintained as an episomal element. Once favorable conditions arise, the phage begins either the lytic or lysogenic cycle. It is thought that this mechanism can lead to persistent infections and might contribute to phage survival during unfavorable growth conditions in natural environments [22,23]. It has been proposed that *Bacteroides* and *Escherichia* species can be infected in this manner in the gut [24,25]. Finally, chronic infections involve the release of virions without killing the infected cell [26]. To our knowledge, the incidence and consequences of chronic bacteriophages in the gut has not been explored.

Phages influence microbial community structure and function through various mechanisms [27,28,29,30,31]. Density-dependent lysis of bacterial species in a microbial community (also known as Lotka-Volterra dynamics [32,33]) promotes microbial diversity and evolution, and maximizes the efficiency of resource usage by the community [27,29,30,31]. On the other hand, lysogeny can influence the community composition through indirect benefits to the bacterial lysogen (prophage carrier bacteria) and through horizontal gene transfer (HGT) of beneficial genes between hosts [18,34,35,36,37,38,39]. Prophage-encoded selective advantages include: (i) protection from superinfection; (ii) release of activated prophages from a subset of the lysogenized population that can subsequently lyse competing species; (iii) prophage-encoded pathogenesis determinants such as toxins or host adherence factors; (iv) genes that increase fitness of the lysogen under certain conditions; and (v) reducing the substrate utilization by its host [13,35]. Unfavorable conditions and low host density are parameters known to primarily select for phages which undergo lysogenic replication, both under laboratory conditions and in complex environments [35,40]. Recently, examination of natural bacterial communities has shown that increasing host densities might select for the strategy of lysogeny as well [41].

**Figure 1:**
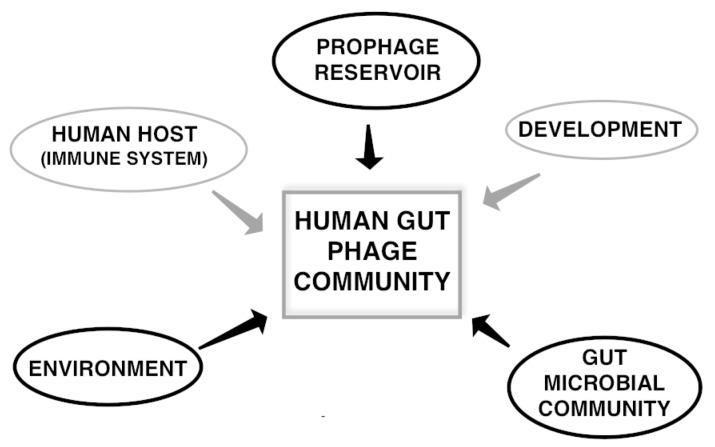
Major factors influencing the structure, function, and dynamics of the gut phage community. Factors that can influence the phage community and serve as a source of phages are marked in thick black circles. Factors that only influence the viral community composition are marked in grey.

To fully understand the role of phages in shaping the gut microbial community structure, one needs to consider four major influences of the system: the human host and its immune system, the structure and function of the microbial community itself, the environmental inputs, and the gut viral community (both eukaryotic and prokaryotic). Both the role of eukaryotic viruses and the interactions between the immune system and viruses have been studied and reviewed elsewhere [42,43,44,45,46,47]. Although the role of the host immune system in shaping the phage community and the role of external factors is not the focus of this review, it is important to note that these factors play an important role as well (Figure 1).

## 3. Development of the Gut Phage Community

**Figure 2:**
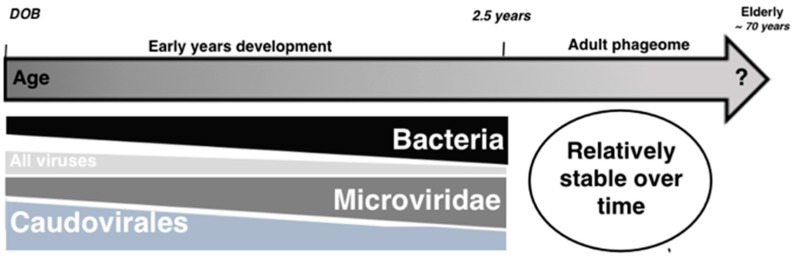
Schematic representation of the gut phage community development with age. Individuals are colonized early after birth, within 0–4 days after birth (DOB). During the first days of life, the diversity of the phage community is high and the microbial community abundance and diversity is low [51]. A reduction in *Caudovirales* diversity leads to an expansion and a shift in the microbial community composition and an increase in *Microviridae* phage diversity and abundance. A relatively stable phage community is maintained during the adult life. Changes in the phage community associated with a shift towards an elderly-like microbial community are unknown.

The microbiome structure of healthy newborns evolves in the first 2–3 years of life from a near sterile environment towards a diverse adult-like microbiome that is maintained throughout adulthood, until age-related changes progressively promote composition changes towards an elderly-like microbiome (Figure 2) [48,49]. Breitbart et al. carried out the first study of infant gut bacteriophages from 1 week to 3 months of age, and showed that the viral diversity in newborns was extremely low and dynamic [50]. More recently, two studies examined the dynamics of viruses and the microbiota during the first 2 and 2.5 years of life, respectively [51,52].

These analyses showed that the gut is colonized by phages quickly after birth (1–4 days), that gut phages are more similar between infants than between adult individuals, and that the phage community undergoes considerable changes early in life [51,52]. There is a slight decrease in double stranded DNA (dsDNA) phage diversity, specifically of *Siphoviridae* phages, and an increase in single stranded DNA (ssDNA) phage diversity in infants compared to adults [51,52]. Lim et al. showed that the phage community richness is highest in the first 1–4 days of life. The authors hypothesize that the community undergoes reverse Lotka-Volterra dynamics, in which the initial high diversity of bacteriophages is not supported by the low microbial abundance, leading to a collapse of the phage diversity and the predatory pressure [51]. Consequently, an increase in the microbial abundance and a shift in the community composition drives a shift in the phage community composition as well [51] (Figure 2). The authors could not determine the provenance of the phages or their lifestyle. They venture that phages might be transmitted from the mother to the baby through the placenta [51]. Another possibility could be the transmission of phages through the mother’s breast-milk, although Breitbart et al. demonstrated that the most abundant phages in infants were not found in breast milk nor formula. An important source of phages early in life is activated prophages from the microbial community [50,53,54]. A recent study identified and analyzed prophages from *Bifidobacterium* species, a dominant member of the infant microbiome, and showed how they can deeply impact the infant gut microbiota development [55]. Little is known about how bacteriophages influence the shift from the adult to the elderly microbiome.

Disruption in the proper gut phage community development has been correlated with a higher risk of disease onset. A study by Reyes et al. compared the virome of healthy twins, and twins discordant for severe acute malnutrition (SAM) during their first 2.5 years of life [52]. The virome in individuals that suffer SAM is significantly less variable during the first years of life than that of healthy individuals. However, only one of the twins showed disease symptoms, suggesting that a healthy phage community development towards an adult-like community is important in maintaining health, but its disruption is not enough to result in disease. However, this work provided a set of valuable viral markers (including both prokaryotic and eukaryotic viruses) that can be used to identify at-risk populations. Deep sequencing and analysis of whole community metagenomes using novel metagenomics methods has proven useful in the characterization of bacterial species at the strain level and associated phages in young infants. Applying these methods to understand health and disease-related phage-host candidates will be valuable to understand the proper development of the gut phage community [53,54].

## 4. Characteristics of the Adult Gut Phage Community

**Figure 3:**
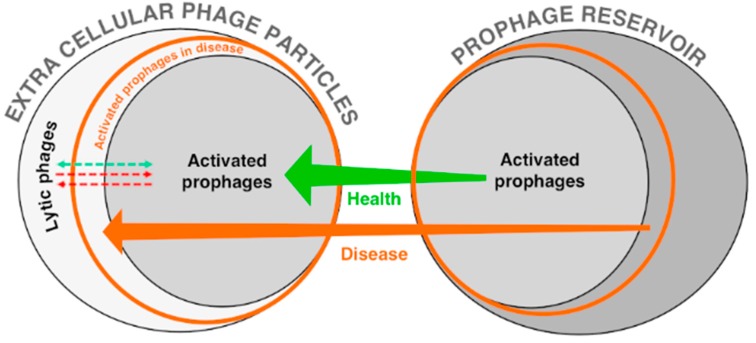
The adult gut phage community in health and disease. Bacteriophages found in human stool are an ensemble of lytic phages and activated prophages that arise from a larger prophage reservoir found in the gut microbial community. It has been proposed that the increase in phage diversity in inflammatory bowel disease patients comes from activation of a larger fraction of the prophage reservoir, which in turn leads to a decrease in bacterial diversity [11]. An increase of lytic phages has also been correlated with disease [60], suggesting that a proper balance between lytic phages and activated prophages is important to maintain health.

The diversity of phages associated with the adult human gut has been examined both through transmission electron microscopy (TEM) of virus particles isolated from stool samples and metagenomic analysis. It is important to note that the virus particles in the feces predominantly represent extracellular temperate phages (prophages that have become activated and lysed their host upon virion release) and lytic phages (Figure 3). For brevity, phage particles found within stool samples will be referred to as EC-phages (phages found as extracellular particles). Recent studies aimed at optimizing phage particles (PPs) extraction from stool samples have reported higher PP counts than previously thought. Individuals have between 10^9^–10^10^ particles per gram of feces (dry weight) compared to 10^11^–10^12^ bacteria [56,57]. In fact, Hoyles et al. hypothesized that due to inefficiency in the purification process, the actual phage concentration in feces could range between 10^10^–10^12^ particles/g of feces [57]. A significant number of these phages are associated with the mucosal gut membrane [58,59]. Even taking into account more accurate PP counts, the virus to microbe ratio (VMR) in the gut is still significantly lower compared to other microbial communities [41]. TEM analysis revealed primarily *Myoviridae*, *Siphoviridae*, and *Podoviridae*-like morphologies characteristic of phages from the *Caudovirales* order [56,57].

Metagenomic analysis of whole community and EC-phages provides a broader understanding of the actual community diversity. EC-phage metagenomes demonstrate the individuality and stability over time of gut phages [61,62,63,64]. The bacteriophage community is dominated by dsDNA and ssDNA phages. The majority of RNA viruses found in the gut are plant and human viruses [65], with only a limited number of RNA gut bacteriophages being described in primate metagenomes [66]. RNA coliphages have been isolated from the gut at very low titers [67], compared to other coliphages [60] or *Bacteroides* phages [24]. Generally, the phage community consists of a few dominant DNA phage types, with a tail of less abundant phages [62]. The most abundant phage types can represent more than 15% of the total phage community [62]. Most of the phages that can be taxonomically classified are from the *Caudovirales* order [56,61,62,63], and the *Microviridae* family.

**Figure 4:**
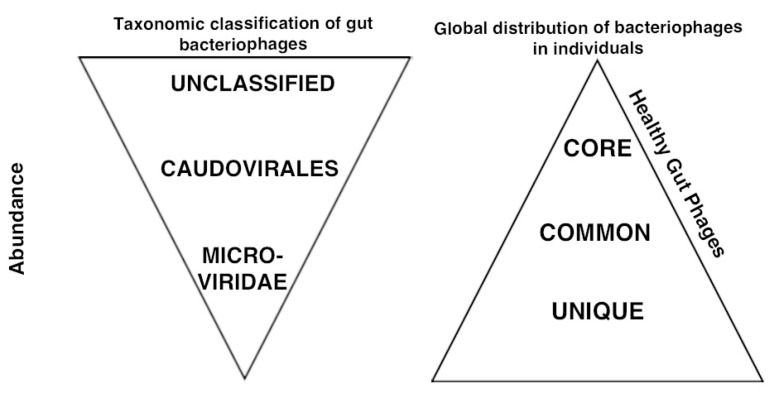
Bacteriophages in adult healthy individuals. The bacteriophage community of adult individuals is composed of members of the *Caudovirales* order, the Microviridae family and a large fraction of unclassified phages. Healthy individuals contained a relatively unique phageome and a set of shared phages between individuals that is globally distributed (<5% of total phageome). This set of shared phages has been correlated with health and proposed as the healthy gut phageome (HGP).

Even though representation of gut phages in public databases is increasing, approximately 50% of the sequences cannot be classified (Figure 4). Methods to overcome the low rate of classified sequences have been developed. For example, phage genome [64,68] and phage protein-cluster analysis [69,70,71], or the recently developed Homologous Virus Diversity Index (HVDX) [72] reduce the data complexity and enhance virus classification of metagenomic datasets. These methods, similar to other analysis pipelines such as PHACCS (Phage Communities From Contig Spectrum) [73], can also be used to analyze the diversity of the phage community. For instance, HVDX determines the diversity of the phage community after genome-based similarity analysis and binning. Overall, this type of analysis reduces the diversity estimates that single contig-based analysis provides [64,68,71,74]. Part of the inability to classify more gut viruses can be explained by the underrepresentation of Firmicutes and *Bacteroides* phages in public databases [19]. Some of the most used metagenomic analysis pipelines for viral metagenomes are MetaVir [75], Virome [76], and Megan [77]. Waller and colleagues attempted to increase the accuracy of phage taxonomic classification and quantification by identifying phage-specific orthologous protein groups [78]. These include several quantitative taxon-specific maker genes that can be used to classify bacteriophage sequences within whole community and EC-phages metagenomes. They were able to identify 15 different bacteriophage taxa (mostly at the genera level) in human gut metagenomes. The most abundant taxa are podovirus, myovirus, and siphovirus, and their abundance varies between individuals [56,57,79].

Analyses which have expanded our knowledge of *Microviridae* phages have increased our appreciation for their importance and abundance in the adult gut phage community [51,52,79,80]. *Microviriade* phages are ssDNA phages, with a small genome size, typically between 4–7 kb that are currently divided into four groups: *Microvirus*, *Pichovirinae*, *Alpavirinae*, and *Gokushovirinae*. The two latter groups are highly associated with the gut microbial community [80]. Despite the initial categorization of *Microviridae* as lytic phages, some have been identified as prophages of *Bacteroides* and *Parabacteroides* species [80,81]. The decrease in the *Microviridae*:*Caudovirales* ratio has been correlated with diseases such as inflammatory bowel disease (IBD), suggesting that *Microviridae* phages are important for health [11]. Importantly, a significant fraction of EC-phages is classified as temperate phages.

Even though the gut phage community is stable over time and is for the most part unique to an individual, global analyses of gut metagenomic datasets across the world have revealed that there is a globally distributed phageome [52,64,82,83,84,85]. On a small scale, it has been observed that individuals that share living spaces tend to have more similar phages [86]. Some of these shared phages are transmitted between household members. Individuals that are not undergoing antibiotic therapy are more frequently the source of these transmissions [87]. Moreover, a recent study analyzing the Earth’s virome showed that 83% of the gut viral sequences were found in at least two individuals, and 30% were found in more than 10% of the metagenomes (148 human stool metagenomes) [82]. A similar trend was observed in more limited studies that support the existence of a reservoir of common phages (Box 1) [52,64,83,84,85]. One of the most globally distributed phages is the novel phage termed crAssphage which was found in 73% of all the fecal metagenomes analyzed (450) [83]. A separate study, aimed at understanding poorly represented phages in EC-phages metagenomes, revealed a group of *Bacteroidales*-like phages that is shared between individuals [84]. Moreover, these phages could be used to classify individuals into four different viral-enterotypes (groups characterized by a certain virus membership profile) based on the abundance profile of these phages in the 139 gut metagenomes analyzed [84]. Overall, the gut phage community is an ensemble of phages with a component that is unique to each individual, and a component that is shared by many individuals (Figure 4). The presence of shared bacteriophages among healthy individuals raises the question of what is their role in maintaining health.

Recently we carried out a study to characterize the extent of a global gut phageome and its implication in health [64], and showed a correlation between shared phages and health status. In this work, we combined ultra-deep sequencing of two healthy individuals with gut phage metagenomic analysis of healthy and diseased individuals [11]. Using 4301 phages found in the two study individuals, a set of 23 “core phages” was identified in more than 50% of healthy individuals from different geographic locations, and a set of 155 “common phages” was found in 20–50% of the individuals. More shared phages were identified in the additional healthy phage metagenomes. Importantly, both the percentage of individuals that carry each core phage and the percentage of total core and common phages that each individual carried were reduced in individuals suffering from IBD. Even though these phages represent only a minor component of the total community (<5%) (Figure 4), their relation to health led us to propose the existence of a Healthy Gut Phageome (HGP; Box 1). The existence of a prophage reservoir in individuals that is maintained over time and serves as an important source for EC-phages (Figure 1) [17,60,85] led us to hypothesize that a fraction or the totality of the HGP in healthy individuals can potentially arise from this reservoir. However, the origin of the HGP and its role and mechanisms in contributing to the healthy structure of the microbiome is unknown and is currently under investigation.

## 5. Towards an Equilibrium between Lysis and Lysogeny

The balance between lysis and lysogeny, and a differential spatial distribution of phages appears to be correlated with health [11,60,88] (Figure 3). Local distribution of phages in the gut is altered in dysbiotic states. Viral communities associated with the lumen and mucosal surfaces differ in healthy mice. A greater increase of temperate *Caudovirales* in mucosal-associated phages compared to luminal phages in obese mice results in the loss of these differences [89]. It has been shown that in leukaemic diseases [60], and in IBD [88], individuals shed a higher number of EC-phages in their feces. This can be due to either higher rates of lytic phage activity or higher prophage induction. Studies by Furuse and colleagues focused exclusively on coliphages showed that most coliphages in leukaemic patients were virulent, as opposed to a dominance in temperate phages in healthy individuals [60]. Additionally, Norman and colleagues hypothesized that the increased phage diversity in IBD patients might be due to the activation of prophages [11]. A combination of whole community and EC-phage metagenomic analysis has been used to determine the proportion of lytic versus temperate phages in stool samples. The percentage of EC-phages which can be classified as temperate ranges between 17–37% [62,85]. Because phage contigs generally represent incomplete phage genomes, these numbers are only a lower bound. Waller et al. analyzed the abundance of whole community phage taxa and prophage-reservoir taxa in 252 fecal metagenomes from 207 individuals, and determined that the combined prophage reservoir of individuals was novel [78]. Moreover, a total of seven out of 15 phage taxa from whole gut community analysis were found as predicted-prophages [78]. Additionally, the relative abundance of temperate phages in the community has been estimated by quantifying the number of metagenomic reads associated with typical-lysogeny genes such as integrases. These estimations depend on multiple parameters, and range from 25–50% of the EC-phages as temperate [85,90]. This approach suggests that the contigs identified as temperate phages comprise a significant percentage of the entire community. Additionally, in vitro studies have shown a high proportion of temperate coliphages as well [60]. Consequently, the dominance of gut bacterial communities by temperate phages has become a paradigm of the field. It has been proposed that the reduction of the VMR ratio in high-density microbial communities can be driven by a suppression of lysis and a switch to lysogeny [41]; however, Weitz and colleagues concluded that there is not enough evidence to support these claims yet, and that further work is needed to characterize the relative abundance and role of lysogeny in shaping dynamics within environmental and human-associated systems [91].

Understanding the balance between lytic phages, activated prophages, and the total prophage reservoir is important in understanding the healthy gut. Mills and colleagues proposed the community shuffling model (Box 1) [17,18], which explains the shift from health to disease through increased induction of certain prophages. Prophage activation is likely sensitive to stress, therefore processes such as inflammation in patients with IBD are likely to result in an increase of prophage activity (Figure 3). Determining activation dynamics of specific prophages during “steady-states” may help to identify what is necessary to bring a community structure back to equilibrium from dysbiotic states. A study aimed at investigating the active portion of the total prophage reservoir and the level of prophage activation showed that: (i) prophage activation varies with time; (ii) approximately 24% of predicted prophages with a known host are active in at least two individuals; (iii) an abundance of prophage-taxa is generally correlated with the abundance of activated prophages; and (iv) a set of 50 active core prophages are found in more than half of the individuals [78]. Furthermore, the activation levels of the 25 most abundant prophages predicted from reference genomes showed no or a low level of induction for ~50% of the prophages, as indicated by the equal abundance of prophage and host DNA (molar ratio 1:1). In ~40% of the cases, the abundance of the prophages was less than the abundance of its host genome (molar ratio < 1), suggesting that the prophage was absent in a portion of the host population. In the remaining 10% of the cases, prophages were considerably more abundant (molar ratio > 1), suggesting prophage induction of a large portion of the population or infection in a different host. These findings are consistent with a similar study previously carried out by Stern et al. [85]. Prophages associated with reference genomes that contained either antibiotic resistance genes or virulence factors were found to be activated at low levels in almost all individuals [78]. These results suggest that prophages with beneficial genes for the host are more likely to be maintained active within the community. Additionally, some prophage taxa were related to multiple host taxa. This implies that the host with which a given prophage is associated might be more important than the presence or absence of the prophage itself. Recent studies have shown molecular mechanisms involved in controlling the switch between lysis and lysogeny at the single cell level [21,23,92]. The importance of a carrier state of the temperate phage as a non-integrated episome to maintain a stable coexistence of a phage and its host has recently been shown [23], adding complexity to the studies of lysogeny. Combining molecular studies with whole community and EC-phage analysis of phages correlated with health and disease will likely contribute significantly to our understanding of the equilibrium of phages during health.

Among the various anti-phage defense systems that bacteria encode, such as restriction modification systems or abortive infections [93,94], the CRISPR/Cas system may play a role in lysogeny [95]. The CRISPR system is a phage defense system found in Archaea and Bacteria. Upon phage infection, a small fragment of the invading phage genome of approximately 30 bp (known as a spacer sequence) becomes incorporated into the bacterial chromosome in a CRISPR array [96]. This spacer sequence is later used to recognize and destroy invading phages. A set of CRISPR spacers targeting temperate phages has been identified [85,96,97]. In some cases, encoding for a spacer sequence against a prophage will lead to cell death [98], however, both elements can occasionally coexist [99]. For instance, recent work done by Goldberg et al. [95] shows that the type III CRISPR/Cas system in *Staphylococcus epidermidis* tolerates lysogenization, but prevents lytic infection and reduces prophage induction through the degradation of transcripts necessary for lytic replication. Stern and colleagues identified 991 spacer-targeted phages in the human gastrointestinal microbiome [85]. Approximately 37% of these were temperate phages and were being targeted by spacers conserved among different individuals, demonstrating the prevalence of prophage-targeting spacers. However, in most cases (85%), if an individual encoded a spacer sequence, its phage target was not present. Thus, the most likely scenario is that the CRISPR system in the gut contributes to the ecology of frequently activated prophages.

Gnotobiotic mice models have proven useful for understanding gut phage dynamics. Duerkop et al. [100] demonstrated that the induction of the prophage provides an advantage to its host by killing competitors, both in vitro and in vivo. De Paepe and colleagues [17] modeled prophage activation and showed that prophage induction in the mouse gut is higher than in vitro, and that it results in a significant cost to its bacterial host. However, the cost of induction is compensated by the initial killing of susceptible cells by the released virions, after which the initial lysogen strain and newly infected cells that have become lysogenized coexist. During their experiment, resistant mutants independent of phage pressure arose, which demonstrates that bacterial selection in vivo depends on a variety of factors. Importantly, they captured the occurrence of HGT, which suggests that the role of phages in promoting HGT can be studied using mouse models. In a separate study, Reyes et al. hypothesized that prophage activation occurs in feces due to nutrient limitation [61]. To test this hypothesis, germ-free mice were colonized with *Marvinbryantia formatexigens* and *Bacteroides thetaiotaomicron,* and RNA transcription was measured to study prophage expression in the cecum and feces. Only one out of three prophages from *M. formatexigens* was fully expressed in feces. In contrast, 50% of the samples from the cecum showed expression of the same prophage. Only genes involved in the maintenance of lysogeny were expressed for the other monitored phage, both in the cecum and feces. Mouse models to study phage-host dynamics of more complex communities have also been developed [101]. Mice colonized with a consortium of 15 human gut bacterial species were challenged with human EC-phages and changes in the host community, EC-phages and prophage activation were simultaneously measured. The study found that human EC-phages could replicate and affect the community structure in the mouse model. In this study, the similarity between the phage relative abundance found in the feces as compared to the cecum depended on the phage type. One of the 15 bacterial species (*Bacteroides cellulosyliticus* WH2) was represented by a library of transposon mutants. As expected, there were no phage mutants in the cI repressor (necessary to control the switch between lysogeny and lysis). Interestingly, phages with mutations in the cI-Rha intergenic region, which facilitate prophage induction, were selected and maintained at high relative abundance, even before prophage induction. These results suggest that prophages might be providing fitness advantages to their host through mechanisms unrelated to “killing competitors.” Importantly, mouse models can also be used to study interactions between lytic phages and the gut microbiota. Recently, Maura and colleagues developed a model to study phage replication in the gut [25,59,102]. Reyes demonstrated that there are spatial differences in the activation of certain prophages [61]. Maura showed the replication of lytic phages differed between small intestine, colon, and feces, and between the lumen and mucosal surfaces as well. Interestingly, they demonstrated that these differences could be attributed to variable cellular microbial states affecting phage susceptibility [59]. We refer the reader to a detailed review on this work carried out by Maura and colleagues [25]. Recently, Santiago-Rodriguez and colleagues developed a chemostat-based gut model that is representative of the bacteriophage community found in feces [103]. Additionally, high-throughput gut models such as gut-on-a-chip that can somewhat mimic the human environment seem promising to unravel bacteriophage-bacteria host dynamics in the gut [104,105,106].

A combination of evidence suggests that lysis does not control the microbial community in a kill-the-winner-like fashion, as is observed in many other microbial environments [38,48]. The stability of the healthy gut phage community, and the presence of a large prophage reservoir within the gut, is indicative of a steady-state system. Some groups have suggested that lytic phages might provide protection to the human host from bacterial pathogens [58]. Barr et al. discovered an innate bacteriophage-mediated immune system in which bacteriophages adhere to mucus (BAM) can confer protection to the underlying human epithelium from pathogens [58,104]. Their later work [104] shows that phages attached to mucus have a reduced diffusion capacity that enables them to kill cells at low abundance more efficiently. They further integrate the role of lytic activity with the high prevalence of lysogeny [90]. Briefly, they propose that lysogeny is important in the lumen and regions with lower mucus concentration, and that constitutes a first layer of protection from bacterial pathogens by providing competitive advantages to their host. If the pathogen is still able to outcompete the commensal organisms and get closer to the epithelial cells, the BAM immunity, which preferentially attacks low abundance members, will destroy the pathogen. It is reasonable to speculate that BAM immunity might affect low abundance commensal members of the microbiome as well, such as Proteobacteria. If this was the case, lysis could contribute to the structure of the community in a kill-the-loser dynamic. However, it is important to keep in mind that BAM immunity has only been demonstrated in a single host species system at relatively low cell concentrations. Overall, these studies highlight the importance of a proper balance between lysogeny and lysis.

Determining the host range of the gut phage community is critical for understanding phage dynamics. The culture-independent advantage that viral metagenomics provides comes at the cost of losing host context. Initially, gut phage–host relationships were predicted through comparison to phages with known hosts [61,62], and comparison of whole-community metagenomes to EC-phage metagenomes. Bioinformatic advances are providing new tools to predict phage–host interactions in microbial communities [107]. For instance, Marbouty et al. presented a novel whole-community metagenomic technology that takes advantage of physical contact between the phage and bacterial host genomes to predict phage hosts [108]. Their results showed that even though phages tend to have a preferred bacterial host, multiple phages were associated with more than one taxonomic group. Additional studies indicate that the host range of some phages in the gut might be much broader than previously thought [39,71,78,82]. Experimentally, some lytic phages and activated prophages within the gut have been shown to have a broad host range [24,60,102,109]. Thus, the gut phage community is likely a collection of viruses representing a spectrum of host ranges from specialist phage-taxa (connected to one or a low number of bacterial taxa), to generalist phage-taxa connected with multiple bacterial-taxa (up to four different phyla) [70,78]. However, observations from bioinformatic analysis of large datasets should be tested experimentally. Understanding the potential and actual host-range of phages under conditions of gut health and disease is needed for advancement of the field.

## 6. Gut Phages, Microbial Resilience, and Health

**Figure 5:**
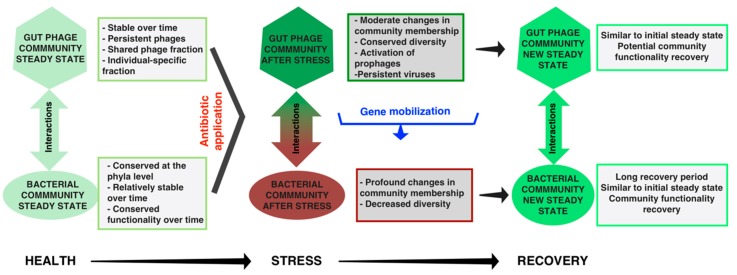
Phage community resilience and its contribution to the resilience of the bacterial community. A characteristic of a healthy microbiome is its ability to recover quickly from perturbations, after which a new “steady state” is established. Antibiotic administration results in profound changes in the microbial community, while changes in the viral community are only moderate. Phage–bacteria network interactions increase after perturbation and new networks are established in the new steady states. The phage component likely contributes to the recovery of the microbial community through the mobilization of beneficial genes.

A hallmark of an adult healthy gut microbiome is its resilience. It has been shown that the phage community may partially contribute to this feature [39] (Figure 5).

After rapid changes early in life, the adult gut phage community remains remarkably stable [61,63,64]. Approximately 80% of the phages are maintained over 2.5 years (duration of study) in an individual [63]. Despite this stability, certain external factors can promote changes in its diversity or and composition. Minot et al. [62] demonstrated that the composition of the virome changes with drastic dietary shifts. Although they did not examine whether it returns to its normal composition after the end of the imposed diet regime, the high phage community stability over 2.5 years observed in their longitudinal study suggests that the phage community is maintained despite minor dietary changes. Howe et al. showed that different diets can promote differential long-term changes in the stability of the mouse phage community and in its interaction network with the microbial community [110]. Thus, long term effects of different diets and treatments on the phage community should be studied and considered when using microbiome-targeted therapeutic treatments to promote health.

The gut microbiota is deeply affected by antibiotic treatment, after which a new, but similar, microbial “steady-state” is restored [4,111] (Figure 5). In contrast, the diversity of the viral community measured through the HVDI index is unaffected despite a shift in the overall viral community membership, and the persistence of certain viral species [87,112]. Overall, individual-specific patterns are maintained and the viral community reaches a new “steady-state” after antibiotic treatment as well [87,112]. Interestingly, an increase of antibiotic-resistance genes in the gut virome has been associated with antibiotic exposure [39,112]. Modi and colleagues demonstrated the HGT-based contribution of the phage fraction to the resilience of the microbiome after antibiotic treatment [39] (see Sun et al. for a detailed review [113]). The functional resilience of a healthy microbiome is not strictly dependent on taxonomic diversity. Multiple gut microbial communities can provide a similar level of functional resilience [3,4,5]. In comparison, due to a high fraction of unknown sequences in gut metagenomes, the functional diversity provided by the phageome is not well understood [61]. Moving towards a more complete understanding of the functionality of the gut phageome is needed. Overall, the potential of phages to contribute to the resilience of the microbiome suggests that phages play a role in maintaining a healthy equilibrium in the gut and are likely influential in the re-establishment of healthy symbiosis from disease.

**Figure 6:**
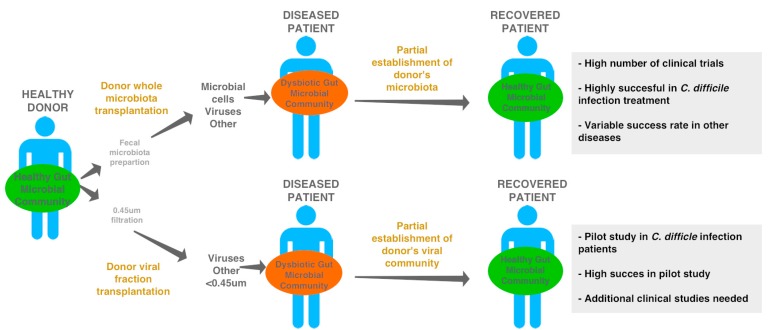
Fecal microbial and the potential of viral filtrate transplant treatment to restore health. Fecal microbial transplants are considered a successful tool to treat certain microbiota-associated diseases and disorders [115,116,117,118,119,120,121,122,123,124,125,126,127]. Recently, a pilot study demonstrated that a transplantation of viral filtrates was sufficient to restore health in *Clostridium difficile* patients. This result highlights the potential for viruses to influence microbial communities, ultimately affecting health and disease.

Before phages were considered as a tool to shift a dysbiotic community back to eubiosis, fecal microbial transplantation (FMT) was used to successfully treat certain diseases [114,115] (Figure 6).

FMT involves the transplantation of feces from a healthy individual to the gut of an individual suffering a gastrointestinal-related disease. FMTs are highly successful in the treatment of *Clostridium difficile* infection (CDI), with recovery rates approaching 95% [116,117,118]. In contrast, the treatment of other disorders, including but not limited to inflammatory bowel disease [119], irritable bowel syndrome [120], and metabolic syndrome [121,122], has variable rates of success [119]. The scientific community is trying to identify predictive markers for the successful establishment of the new microbiota that leads to an effective treatment.

The role of phages in the re-establishment of health during FMT treatments has received little attention thus far. To our knowledge, only two studies have followed the establishment of bacteriophages during FMT treatments. Broecker and colleagues followed a CDI patient who had received FMT treatment for four and half years [123,124,125]. Microbial community DNA was sequenced and approximately 10 different bacteriophage types were detected [125]. Further analysis revealed the presence of 22 viruses throughout donor and patient samples. Most of the phages were found only in one sample, but some were successfully transferred from the donor to the patient [124]. Chehoud et al. analyzed the EC-phage community of a single donor and three ulcerative colitis patients that had received FMT [126]. The successful transfer of 32 different donor viral contigs to FMT recipients was reported. Moreover, they showed that phages from the *Siphoviridae* family (which tend to be temperate) were more efficiently transmitted than phages from other taxonomic groups. This result suggests that temperate phages might have a competitive advantage over others, either by having higher host range, higher host availability, or by being transmitted together with their host as prophages. Analysis of the phage component in a diversity of FMT trials with different success rates might be able to explain variations among trials. Thus, it is important to consider phages as a key component of the microbiome in further FMT analysis. Interestingly, risperidone-induced weight gain can be reproduced in mice through transplantation of the fecal phage fraction alone, which suggests that healthy phage community transplantation might have positive effects as well [127].

A recent pilot study has shown that the gut phage community by itself may be sufficient to eliminate CDI and promote the recovery of a healthy microbiome structure [128] (Box 1; Figure 6). In this study, fecal filtrate transplants (FFT), in which cells are removed but smaller particles such as viruses are retained, were effective in the treatment of five patients suffering from CDI. In all the cases, FFT restored normal stool habits and eliminated CDI symptoms for at least six months. Although only the viral community of one donor and one recipient was analyzed through metagenomics, high similarity between their viral community suggests the successful establishment of microbiome-associated phages through FFT. Caution is necessary in the interpretation of these results, since other variables, such as other mobile elements, might be responsible for the changes. Overall, these results highlight that bacteriophages alone might be able to shape the structure of the microbial community and serve as therapeutic agents to restore and maintain health. Future clinical studies are needed to determine the role of phages in restoring health and to increase our knowledge on the role of phages in human health.

## 7. Remarks and Future Directions

It is becoming clear that bacteriophages contribute to the human gut microbial community structure and function, ultimately influencing states of health and disease. The proper development and structure of the gut phage community is likely important in maintaining health [11,52], suggesting that the concept of microbial dysbiosis can be extended to the phage component of the microbiome. Many of the bacteriophages present in the gut arise from the induction of prophages present in the resident bacterial community [17]. However, only a fraction of the total prophage reservoir in healthy individuals can be found as EC-particles [78]. Preliminary studies suggest that the more active prophages tend to encode beneficial genes for their bacterial host. Determining what factors influence prophage activation from the large bacterial reservoir, during healthy and dysbiotic states, will shed light onto mechanisms by which the gut phage community impacts health and disease. The influence of lytic phages in the gut has received less attention [25], but it has been suggested that they may significantly contribute to the protection of the human host from pathogen colonization [58,90].

Currently, the study of gut viral ecology is primarily in a descriptive phase, in which basic ecological parameters such as diversity, spatial distribution, and connectivity between community members are being determined in different human populations of varying health states. In the near future, it is important that the field continues to move beyond studies of correlation and further investigate the direct impacts of gut viruses during health and disease. Mouse model systems, artificial consortiums of gut microbial communities, bacterial genome editing, and novel bioinformatic analysis of metagenomic datasets, bioreactors, and ex vivo model systems can be used to this end. Ecology-driven studies should be performed as well. Constant external inputs can lead to stochastic events within this “internalized external” environment. Determining stable associations between microbial species and the human gut as opposed to transient microbial species will increase our understanding of associations important to human health. Isolating representative phages from the human gut is necessary to develop useful models to be able to study the molecular mechanisms of bacteria-phage interactions in the gut [129]. Additionally, the spatial distribution of phage infection in health and disease as well as the temporal co-evolution of phages and their hosts in vivo will provide important information on the ecology of bacteriophages in the gut. Continued efforts among researchers to generate curated databases with reference phage genomes and host genomes will allow for a more systematic investigation of host-virus interactions. Further incorporation of computational models to investigate host-virus dynamics will help advance our understanding of viral gut ecology.

The possibility of a widely-distributed gut phageome among healthy individuals has important implications for disease treatment. We propose that the healthy phage community may be sufficient to return a dysbiotic community to a healthy “steady-state”. Preliminary evidence showing both the contribution of the phage community to the resilience of the microbiome [39], and the recovery of diseased individuals through fecal filtrate transplants [128], provide exciting evidence for this proposal. We anticipate the chemostat and mouse models that mimic the gut community will be useful to determine to what extent phages can be used to manipulate the microbiota structure, and for testing mixtures of microbes that can be used in fecal transplant treatments to decrease risks associated with such practices [130]. The mechanisms by which phages influence microbial community structure and function in the healthy and diseased gut microbial community is an exciting area of research.

Box 1 **Key concepts and definitions of the human gut phage community.**

**Gut phages and health:** gut phages play a role in shaping the gut microbiome structure and function, ultimately affecting health and disease.

**Healthy gut phages:** phages shared among healthy individuals, and whose presence has been correlated with health.

**Prophage reservoir:** all the prophages encoded in normal members of the gut microbial community.

**Resilience**: the capacity of a community to recover from a perturbation and return to a new steady-state.

**Community shuffling model:** a shift in activated prophages found as extracellular phage particles can contribute to a shift of the microbial community, ultimately leading to dysbiosis and disease.

**Potential clinical applications:** phages may be used to re-establish a healthy microbial community structure and recover health.

## References

[1] Lloyd-Price J., Abu-Ali G., Huttenhower C. (2016). The healthy human microbiome. Genome Med..

[2] Faith J.J., Guruge J.L., Charbonneau M., Subramanian S., Seedorf H., Goodman A.L., Clemente J.C., Knight R., Heath A.C., Leibel R.L. (2013). The long-term stability of the human gut microbiota. Science.

[3] Backhed F., Fraser C.M., Ringel Y., Sanders M.E., Sartor R.B., Sherman P.M., Versalovic J., Young V., Finlay B.B. (2012). Defining a healthy human gut microbiome: Current concepts, future directions, and clinical applications. Cell Host Microbe.

[4] Dethlefsen L., Huse S., Sogin M.L., Relman D.A. (2008). The pervasive effects of an antibiotic on the human gut microbiota, as revealed by deep 16S rRNA sequencing. PLoS Biol..

[5] Relman D.A. (2012). The human microbiome: Ecosystem resilience and health. Nutr. Rev..

[6] Human Microbiome Project (2012). Structure, function and diversity of the healthy human microbiome. Nature.

[7] Qin J., Li R., Raes J., Arumugam M., Burgdorf K.S., Manichanh C., Nielsen T., Pons N., Levenez F., Yamada T. (2010). A human gut microbial gene catalogue established by metagenomic sequencing. Nature.

[8] Turnbaugh P.J., Hamady M., Yatsunenko T., Cantarel B.L., Duncan A., Ley R.E., Sogin M.L., Jones W.J., Roe B.A., Affourtit J.P. (2009). A core gut microbiome in obese and lean twins. Nature.

[9] Peterson D.A., Frank D.N., Pace N.R., Gordon J.I. (2008). Metagenomic approaches for defining the pathogenesis of inflammatory bowel diseases. Cell Host Microbe.

[10] Clemente J.C., Ursell L.K., Parfrey L.W., Knight R. (2012). The impact of the gut microbiota on human health: An integrative view. Cell.

[11] Norman J.M., Handley S.A., Baldridge M.T., Droit L., Liu C.Y., Keller B.C., Kambal A., Monaco C.L., Zhao G., Fleshner P. (2015). Disease-specific alterations in the enteric virome in inflammatory bowel disease. Cell.

[12] Sulakvelidze A. (2005). Phage therapy: An attractive option for dealing with antibiotic-resistant bacterial infections. Drug Discov. Today.

[13] Brussow H., Canchaya C., Hardt W.D. (2004). Phages and the evolution of bacterial pathogens: From genomic rearrangements to lysogenic conversion. Microbiol. Mol. Biol. Rev..

[14] Wagner P.L., Waldor M.K. (2002). Bacteriophage control of bacterial virulence. Infect. Immun..

[15] Fuhrman J.A. (1999). Marine viruses and their biogeochemical and ecological effects. Nature.

[16] Reyes A., Semenkovich N.P., Whiteson K., Rohwer F., Gordon J.I. (2012). Going viral: Next-generation sequencing applied to phage populations in the human gut. Nat. Rev. Microbiol..

[17] De Paepe M., Leclerc M., Tinsley C.R., Petit M.A. (2014). Bacteriophages: An underestimated role in human and animal health?. Front. Cell Infect. Microbiol..

[18] Mills S., Shanahan F., Stanton C., Hill C., Coffey A., Ross R.P. (2013). Movers and shakers: Influence of bacteriophages in shaping the mammalian gut microbiota. Gut Microbes.

[19] Ogilvie L.A., Jones B.V. (2015). The human gut virome: A multifaceted majority. Front. Microbiol..

[20] Dalmasso M., Hill C., Ross R.P. (2014). Exploiting gut bacteriophages for human health. Trends Microbiol..

[21] Zeng L., Skinner S.O., Zong C., Sippy J., Feiss M., Golding I. (2010). Decision making at a subcellular level determines the outcome of bacteriophage infection. Cell.

[22] Los M., Wegrzyn G. (2012). Pseudolysogeny. Adv. Virus. Res..

[23] Cenens W., Makumi A., Govers S.K., Lavigne R., Aertsen A. (2015). Viral Transmission Dynamics at Single-Cell Resolution Reveal Transiently Immune Subpopulations Caused by a Carrier State Association. PLoS Genet..

[24] Kai M., Watanabe S., Furuse K., Ozawa A. (1985). Bacteroides bacteriophages isolated from human feces. Microbiol. Immunol..

[25] Maura D., Debarbieux L. (2012). On the interactions between virulent bacteriophages and bacteria in the gut. Bacteriophage.

[26] Smeal S.W., Schmitt M.A., Pereira R.R., Prasad A., Fisk J.D. (2017). Simulation of the M13 life cycle I: Assembly of a genetically-structured deterministic chemical kinetic simulation. Virology.

[27] Rodriguez-Valera F., Martin-Cuadrado A.B., Rodriguez-Brito B., Pasic L., Thingstad T.F., Rohwer F., Mira A. (2009). Explaining microbial population genomics through phage predation. Nat. Rev. Microbiol..

[28] Brum J.R., Sullivan M.B. (2015). Rising to the challenge: Accelerated pace of discovery transforms marine virology. Nat. Rev. Microbiol..

[29] Pal C., Macia M.D., Oliver A., Schachar I., Buckling A. (2007). Coevolution with viruses drives the evolution of bacterial mutation rates. Nature.

[30] Scanlan P.D., Hall A.R., Blackshields G., Friman V.P., Davis M.R., Goldberg J.B., Buckling A. (2015). Coevolution with bacteriophages drives genome-wide host evolution and constrains the acquisition of abiotic-beneficial mutations. Mol. Biol. Evol..

[31] Scanlan P.D. (2017). Bacteria-Bacteriophage Coevolution in the Human Gut: Implications for Microbial Diversity and Functionality. Trends Microbiol..

[32] Lotka A.J. (1927). Fluctuations in the abundance of a species considered mathematically. Nature.

[33] Volterra V. (1926). Fluctuations in the abundance of a species considered mathematically. Nature.

[34] Williams H.T. (2013). Phage-induced diversification improves host evolvability. BMC Evol. Biol..

[35] Paul J.H. (2008). Prophages in marine bacteria: Dangerous molecular time bombs or the key to survival in the seas?. ISME J..

[36] Breitbart M. (2012). Marine viruses: Truth or dare. Ann. Rev. Mar. Sci..

[37] Jiang S.C., Paul J.H. (1998). Gene transfer by transduction in the marine environment. Appl. Environ. Microbiol..

[38] Rodriguez-Brito B., Li L., Wegley L., Furlan M., Angly F., Breitbart M., Buchanan J., Desnues C., Dinsdale E., Edwards R. (2010). Viral and microbial community dynamics in four aquatic environments. ISME J..

[39] Modi S.R., Lee H.H., Spina C.S., Collins J.J. (2013). Antibiotic treatment expands the resistance reservoir and ecological network of the phage metagenome. Nature.

[40] Brum J.R., Hurwitz B.L., Schofield O., Ducklow H.W., Sullivan M.B. (2016). Seasonal time bombs: Dominant temperate viruses affect Southern Ocean microbial dynamics. ISME J..

[41] Knowles B., Silveira C.B., Bailey B.A., Barott K., Cantu V.A., Cobian-Guemes A.G., Coutinho F.H., Dinsdale E.A., Felts B., Furby K.A. (2016). Lytic to temperate switching of viral communities. Nature.

[42] Cadwell K. (2015). The virome in host health and disease. Immunity.

[43] Cadwell K. (2015). Expanding the role of the virome: Commensalism in the gut. J. Virol..

[44] Mokili J.L., Rohwer F., Dutilh B.E. (2012). Metagenomics and future perspectives in virus discovery. Curr. Opin. Virol..

[45] Wylie K.M., Mihindukulasuriya K.A., Zhou Y., Sodergren E., Storch G.A., Weinstock G.M. (2014). Metagenomic analysis of double-stranded DNA viruses in healthy adults. BMC Biol..

[46] Robinson C.M., Pfeiffer J.K. (2014). Viruses and the Microbiota. Annu. Rev. Virol..

[47] Duerkop B.A., Hooper L.V. (2013). Resident viruses and their interactions with the immune system. Nat. Immunol..

[48] Yatsunenko T., Rey F.E., Manary M.J., Trehan I., Dominguez-Bello M.G., Contreras M., Magris M., Hidalgo G., Baldassano R.N., Anokhin A.P. (2012). Human gut microbiome viewed across age and geography. Nature.

[49] O’Toole P.W., Jeffery I.B. (2015). Gut microbiota and aging. Science.

[50] Breitbart M., Haynes M., Kelley S., Angly F., Edwards R.A., Felts B., Mahaffy J.M., Mueller J., Nulton J., Rayhawk S. (2008). Viral diversity and dynamics in an infant gut. Res. Microbiol..

[51] Lim E.S., Zhou Y., Zhao G., Bauer I.K., Droit L., Ndao I.M., Warner B.B., Tarr P.I., Wang D., Holtz L.R. (2015). Early life dynamics of the human gut virome and bacterial microbiome in infants. Nat. Med..

[52] Reyes A., Blanton L.V., Cao S., Zhao G., Manary M., Trehan I., Smith M.I., Wang D., Virgin H.W., Rohwer F. (2015). Gut DNA viromes of Malawian twins discordant for severe acute malnutrition. Proc. Natl. Acad. Sci. USA.

[53] Morowitz M.J., Denef V.J., Costello E.K., Thomas B.C., Poroyko V., Relman D.A., Banfield J.F. (2011). Strain-resolved community genomic analysis of gut microbial colonization in a premature infant. Proc. Natl. Acad. Sci. USA.

[54] Sharon I., Morowitz M.J., Thomas B.C., Costello E.K., Relman D.A., Banfield J.F. (2013). Time series community genomics analysis reveals rapid shifts in bacterial species, strains, and phage during infant gut colonization. Genome. Res..

[55] Lugli G.A., Milani C., Turroni F., Tremblay D., Ferrario C., Mancabelli L., Duranti S., Ward D.V., Ossiprandi M.C., Moineau S. (2016). Prophages of the genus *Bifidobacterium* as modulating agents of the infant gut microbiota. Environ. Microbiol..

[56] Castro-Mejia J.L., Muhammed M.K., Kot W., Neve H., Franz C.M., Hansen L.H., Vogensen F.K., Nielsen D.S. (2015). Optimizing protocols for extraction of bacteriophages prior to metagenomic analyses of phage communities in the human gut. Microbiome.

[57] Hoyles L., McCartney A.L., Neve H., Gibson G.R., Sanderson J.D., Heller K.J., van Sinderen D. (2014). Characterization of virus-like particles associated with the human faecal and caecal microbiota. Res. Microbiol..

[58] Barr J.J., Auro R., Furlan M., Whiteson K.L., Erb M.L., Pogliano J., Stotland A., Wolkowicz R., Cutting A.S., Doran K.S. (2013). Bacteriophage adhering to mucus provide a non-host-derived immunity. Proc. Natl. Acad. Sci. USA.

[59] Maura D., Galtier M., Le Bouguenec C., Debarbieux L. (2012). Virulent bacteriophages can target O104:H4 enteroaggregative *Escherichia coli* in the mouse intestine. Antimicrob. Agents Chemother..

[60] Furuse K., Osawa S., Kawashiro J., Tanaka R., Ozawa A., Sawamura S., Yanagawa Y., Nagao T., Watanabe I. (1983). Bacteriophage distribution in human faeces: Continuous survey of healthy subjects and patients with internal and leukaemic diseases. J. Gen. Virol..

[61] Reyes A., Haynes M., Hanson N., Angly F.E., Heath A.C., Rohwer F., Gordon J.I. (2010). Viruses in the faecal microbiota of monozygotic twins and their mothers. Nature.

[62] Minot S., Sinha R., Chen J., Li H., Keilbaugh S.A., Wu G.D., Lewis J.D., Bushman F.D. (2011). The human gut virome: Inter-individual variation and dynamic response to diet. Genome Res..

[63] Minot S., Bryson A., Chehoud C., Wu G.D., Lewis J.D., Bushman F.D. (2013). Rapid evolution of the human gut virome. Proc. Natl. Acad. Sci. USA.

[64] Manrique P., Bolduc B., Walk S.T., van der Oost J., de Vos W.M., Young M.J. (2016). Healthy human gut phageome. Proc. Natl. Acad. Sci. USA.

[65] Zhang T., Breitbart M., Lee W.H., Run J.Q., Wei C.L., Soh S.W., Hibberd M.L., Liu E.T., Rohwer F., Ruan Y. (2006). RNA viral community in human feces: Prevalence of plant pathogenic viruses. PLoS Biol..

[66] Krishnamurthy S.R., Janowski A.B., Zhao G., Barouch D., Wang D. (2016). Hyperexpansion of RNA Bacteriophage Diversity. PLoS Biol..

[67] Havelaar A.H., Furuse K., Hogeboom W.M. (1986). Bacteriophages and indicator bacteria in human and animal faeces. J. Appl. Bacteriol..

[68] Bolduc B., Wirth J.F., Mazurie A., Young M.J. (2015). Viral assemblage composition in Yellowstone acidic hot springs assessed by network analysis. ISME J..

[69] Lima-Mendez G., Van Helden J., Toussaint A., Leplae R. (2008). Reticulate representation of evolutionary and functional relationships between phage genomes. Mol. Biol. Evol..

[70] Roux S., Enault F., Hurwitz B.L., Sullivan M.B. (2015). VirSorter: Mining viral signal from microbial genomic data. PeerJ.

[71] Roux S., Hallam S.J., Woyke T., Sullivan M.B. (2015). Viral dark matter and virus-host interactions resolved from publicly available microbial genomes. Elife.

[72] Santiago-Rodriguez T.M., Ly M., Bonilla N., Pride D.T. (2015). The human urine virome in association with urinary tract infections. Front. Microbiol..

[73] Angly F., Rodriguez-Brito B., Bangor D., McNairnie P., Breitbart M., Salamon P., Felts B., Nulton J., Mahaffy J., Rohwer F. (2005). PHACCS, an online tool for estimating the structure and diversity of uncultured viral communities using metagenomic information. BMC Bioinformatics.

[74] Ignacio-Espinoza J.C., Solonenko S.A., Sullivan M.B. (2013). The global virome: not as big as we thought?. Curr. Opin. Virol..

[75] Roux S., Tournayre J., Mahul A., Debroas D., Enault F. (2014). Metavir 2: New tools for viral metagenome comparison and assembled virome analysis. BMC Bioinformatics.

[76] Wommack K.E., Bhavsar J., Polson S.W., Chen J., Dumas M., Srinivasiah S., Furman M., Jamindar S., Nasko D.J. (2012). VIROME: A standard operating procedure for analysis of viral metagenome sequences. Stand Genomic Sci..

[77] Huson D.H., Beier S., Flade I., Gorska A., El-Hadidi M., Mitra S., Ruscheweyh H.J., Tappu R. (2016). MEGAN Community Edition - Interactive Exploration and Analysis of Large-Scale Microbiome Sequencing Data. PLoS Comput. Biol..

[78] Waller A.S., Yamada T., Kristensen D.M., Kultima J.R., Sunagawa S., Koonin E.V., Bork P. (2014). Classification and quantification of bacteriophage taxa in human gut metagenomes. ISME J..

[79] Kim M.S., Park E.J., Roh S.W., Bae J.W. (2011). Diversity and abundance of single-stranded DNA viruses in human feces. Appl. Environ. Microbiol..

[80] Roux S., Krupovic M., Poulet A., Debroas D., Enault F. (2012). Evolution and diversity of the *Microviridae* viral family through a collection of 81 new complete genomes assembled from virome reads. PLoS ONE.

[81] Krupovic M., Forterre P. (2011). *Microviridae* goes temperate: Microvirus-related proviruses reside in the genomes of Bacteroidetes. PLoS ONE.

[82] Paez-Espino D., Eloe-Fadrosh E.A., Pavlopoulos G.A., Thomas A.D., Huntemann M., Mikhailova N., Rubin E., Ivanova N.N., Kyrpides N.C. (2016). Uncovering Earth’s virome. Nature.

[83] Dutilh B.E., Cassman N., McNair K., Sanchez S.E., Silva G.G., Boling L., Barr J.J., Speth D.R., Seguritan V., Aziz R.K. (2014). A highly abundant bacteriophage discovered in the unknown sequences of human faecal metagenomes. Nat. Commun..

[84] Ogilvie L.A., Bowler L.D., Caplin J., Dedi C., Diston D., Cheek E., Taylor H., Ebdon J.E., Jones B.V. (2013). Genome signature-based dissection of human gut metagenomes to extract subliminal viral sequences. Nat. Commun..

[85] Stern A., Mick E., Tirosh I., Sagy O., Sorek R. (2012). CRISPR targeting reveals a reservoir of common phages associated with the human gut microbiome. Genome Research.

[86] Robles-Sikisaka R., Ly M., Boehm T., Naidu M., Salzman J., Pride D.T. (2013). Association between living environment and human oral viral ecology. ISME J..

[87] Ly M., Jones M.B., Abeles S.R., Santiago-Rodriguez T.M., Gao J., Chan I.C., Ghose C., Pride D.T. (2016). Transmission of viruses via our microbiomes. Microbiome.

[88] Lepage P., Colombet J., Marteau P., Sime-Ngando T., Dore J., Leclerc M. (2008). Dysbiosis in inflammatory bowel disease: A role for bacteriophages?. Gut.

[89] Kim M.S., Bae J.W. (2016). Spatial disturbances in altered mucosal and luminal gut viromes of diet-induced obese mice. Environ. Microbiol..

[90] Silveira C.B., Rowler F.L. (2016). Piggy-back-the-Winner in host-associated microbial communities. Biofilms Microbiomes.

[91] Weitz J., Beckett S.J., Brum J.R., Cael B.B., Dushoff J. (2016). Lysis, Lysogeny, and Virus-Microbe Ratios. Biorxiv.

[92] Erez Z., Steinberger-Levy I., Shamir M., Doron S., Stokar-Avihail A., Peleg Y., Melamed S., Leavitt A., Savidor A., Albeck S. (2017). Communication between viruses guides lysis-lysogeny decisions. Nature.

[93] Mirzaei M.K., Maurice C.F. (2017). Menage a trois in the human gut: Interactions between host, bacteria and phages. Nat. Rev. Microbiol..

[94] Labrie S.J., Samson J.E., Moineau S. (2010). Bacteriophage resistance mechanisms. Nat. Rev. Microbiol..

[95] Goldberg G.W., Jiang W., Bikard D., Marraffini L.A. (2014). Conditional tolerance of temperate phages via transcription-dependent CRISPR-Cas targeting. Nature.

[96] Sorek R., Lawrence C.M., Wiedenheft B. (2013). CRISPR-mediated adaptive immune systems in bacteria and archaea. Annu. Rev. Biochem..

[97] Mick E., Stern A., Sorek R. (2013). Holding a grudge: Persisting anti-phage CRISPR immunity in multiple human gut microbiomes. RNA Biol..

[98] Edgar R., Qimron U. (2010). The Escherichia coli CRISPR system protects from lambda lysogenization, lysogens, and prophage induction. J. Bacteriol..

[99] Bondy-Denomy J., Pawluk A., Maxwell K.L., Davidson A.R. (2013). Bacteriophage genes that inactivate the CRISPR/Cas bacterial immune system. Nature.

[100] Duerkop B.A., Clements C.V., Rollins D., Rodrigues J.L., Hooper L.V. (2012). A composite bacteriophage alters colonization by an intestinal commensal bacterium. Proc. Natl. Acad. Sci. USA.

[101] Reyes A., Wu M., McNulty N.P., Rohwer F.L., Gordon J.I. (2013). Gnotobiotic mouse model of phage-bacterial host dynamics in the human gut. Proc. Natl. Acad. Sci. USA.

[102] Maura D., Morello E., du Merle L., Bomme P., Le Bouguenec C., Debarbieux L. (2012). Intestinal colonization by enteroaggregative *Escherichia coli* supports long-term bacteriophage replication in mice. Environ. Microbiol..

[103] Santiago-Rodriguez T.M., Ly M., Daigneault M.C., Brown I.H., McDonald J.A., Bonilla N., Vercoe E.A., Pride D.T. (2015). Chemostat culture systems support diverse bacteriophage communities from human feces. Microbiome.

[104] Barr J.J., Auro R., Sam-Soon N., Kassegne S., Peters G., Bonilla N., Hatay M., Mourtada S., Bailey B., Youle M. (2015). Subdiffusive motion of bacteriophage in mucosal surfaces increases the frequency of bacterial encounters. Proc. Natl. Acad. Sci. USA.

[105] Kim H.J., Li H., Collins J.J., Ingber D.E. (2016). Contributions of microbiome and mechanical deformation to intestinal bacterial overgrowth and inflammation in a human gut-on-a-chip. Proc. Natl. Acad. Sci. USA.

[106] McDonald J.A., Fuentes S., Schroeter K., Heikamp-deJong I., Khursigara C.M., de Vos W.M., Allen-Vercoe E. (2015). Simulating distal gut mucosal and luminal communities using packed-column biofilm reactors and an in vitro chemostat model. J. Microbiol. Methods.

[107] Edwards R.A., McNair K., Faust K., Raes J., Dutilh B.E. (2016). Computational approaches to predict bacteriophage-host relationships. FEMS Microbiol. Rev..

[108] Marbouty M., Baudry L., Cournac A., Koszul R. (2017). Scaffolding bacterial genomes and probing host-virus interactions in gut microbiome by proximity ligation (chromosome capture) assay. Sci. Adv..

[109] Kilic A.O., Pavlova S.I., Alpay S., Kilic S.S., Tao L. (2001). Comparative study of vaginal *Lactobacillus* phages isolated from women in the United States and Turkey: Prevalence, morphology, host range, and DNA homology. Clin. Diagn. Lab. Immunol..

[110] Howe A., Ringus D.L., Williams R.J., Choo Z.N., Greenwald S.M., Owens S.M., Coleman M.L., Meyer F., Chang E.B. (2016). Divergent responses of viral and bacterial communities in the gut microbiome to dietary disturbances in mice. ISME J..

[111] Francino M.P. (2015). Antibiotics and the Human Gut Microbiome: Dysbioses and Accumulation of Resistances. Front. Microbiol..

[112] Abeles S.R., Ly M., Santiago-Rodriguez T.M., Pride D.T. (2015). Effects of Long Term Antibiotic Therapy on Human Oral and Fecal Viromes. PLoS ONE.

[113] Sun C.L., Relman D.A. (2013). Microbiota’s ‘little helpers’: Bacteriophages and antibiotic-associated responses in the gut microbiome. Genome Biol..

[114] Bojanova D.P., Bordenstein S.R. (2016). Fecal Transplants: What Is Being Transferred?. PLoS Biol..

[115] Groen A.K., Nieuwdorp M. (2017). An evaluation of the therapeutic potential of fecal microbiota transplantation to treat infectious and metabolic diseases. EMBO Mol. Med..

[116] Fuentes S., van Nood E., Tims S., Heikamp-de Jong I., ter Braak C.J., Keller J.J., Zoetendal E.G., de Vos W.M. (2014). Reset of a critically disturbed microbial ecosystem: Faecal transplant in recurrent *Clostridium difficile* infection. ISME J..

[117] van Nood E., Vrieze A., Nieuwdorp M., Fuentes S., Zoetendal E.G., de Vos W.M., Visser C.E., Kuijper E.J., Bartelsman J.F., Tijssen J.G. (2013). Duodenal infusion of donor feces for recurrent *Clostridium difficile*. N. Engl. J. Med..

[118] Moelling K., Broecker F. (2016). Fecal microbiota transplantation to fight *Clostridium difficile* infections and other intestinal diseases. Bacteriophage.

[119] Gupta S., Allen-Vercoe E., Petrof E.O. (2016). Fecal microbiota transplantation: In perspective. Therap. Adv. Gastroenterol..

[120] Andrews P.J., Borody T.J. (1993). “Putting back the bugs”: Bacterial treatment relieves chronic constipation and symptoms of irritable bowel syndrome. Med. J. Aust..

[121] Li S.S., Zhu A., Benes V., Costea P.I., Hercog R., Hildebrand F., Huerta-Cepas J., Nieuwdorp M., Salojarvi J., Voigt A.Y. (2016). Durable coexistence of donor and recipient strains after fecal microbiota transplantation. Science.

[122] Vrieze A., Van Nood E., Holleman F., Salojarvi J., Kootte R.S., Bartelsman J.F., Dallinga-Thie G.M., Ackermans M.T., Serlie M.J., Oozeer R. (2012). Transfer of intestinal microbiota from lean donors increases insulin sensitivity in individuals with metabolic syndrome. Gastroenterology.

[123] Broecker F., Klumpp J., Moelling K. (2016). Long-term microbiota and virome in a Zurich patient after fecal transplantation against *Clostridium difficile* infection. Ann. N. Y. Acad. Sci..

[124] Broecker F., Klumpp J., Schuppler M., Russo G., Biedermann L., Hombach M., Rogler G., Moelling K. (2016). Long-term changes of bacterial and viral compositions in the intestine of a recovered *Clostridium difficile* patient after fecal microbiota transplantation. Cold Spring Harb. Mol. Case Stud..

[125] Broecker F., Kube M., Klumpp J., Schuppler M., Biedermann L., Hecht J., Hombach M., Keller P.M., Rogler G., Moelling K. (2013). Analysis of the intestinal microbiome of a recovered *Clostridium difficile* patient after fecal transplantation. Digestion.

[126] Chehoud C., Dryga A., Hwang Y., Nagy-Szakal D., Hollister E.B., Luna R.A., Versalovic J., Kellermayer R., Bushman F.D. (2016). Transfer of Viral Communities between Human Individuals during Fecal Microbiota Transplantation. MBio.

[127] Bahra S.M., Weidemann B.J., Castro A.N., Walsh J.W., deLeon O., Burnett C.M., Pearson N.A., Murry D.J., Grobe J.L., Kirby J.R. (2015). Risperidone-induced weight gain is mediated through shifts in the gut microbiome and suppression of energy expenditure. EBioMedicine.

[128] Ott S.J., Waetzig G.H., Rehman A., Moltzau-Anderson J., Bharti R., Grasis J.A., Cassidy L., Tholey A., Fickenscher H., Seegert D. (2017). Efficacy of Sterile Fecal Filtrate Transfer for Treating Patients with *Clostridium difficile* Infection. Gastroenterology.

[129] Koskella B., Parr N. (2015). The evolution of bacterial resistance against bacteriophages in the horse chestnut phyllosphere is general across both space and time. Philos. Trans. R. Soc. Lond. B..

[130] Petrof E.O., Gloor G.B., Vanner S.J., Weese S.J., Carter D., Daigneault M.C., Brown E.M., Schroeter K., Allen-Vercoe E. (2013). Stool substitute transplant therapy for the eradication of *Clostridium difficile* infection: ‘RePOOPulating’ the gut. Microbiome.

